# Effect of dialysate potassium and lactate on serum potassium and bicarbonate concentrations during daily hemodialysis at low dialysate flow rates

**DOI:** 10.1186/s12882-019-1450-7

**Published:** 2019-07-09

**Authors:** John K. Leypoldt, Michael A. Kraus, Bertrand L. Jaber, Eric D. Weinhandl, Allan J. Collins

**Affiliations:** 1 Unaffiliated, San Clemente, California, USA; 2 NxStage Medical, Lawrence, MA USA; 3 St. Elizabeth’s Medical Center, Boston, MA USA; 4 Department of Pharmaceutical Care and Health Systems, University of Minnesota, Minneapolis, MN USA; 5 Medical School, University of Minnesota, Minneapolis, MN USA

**Keywords:** Bicarbonate, Daily dialysis, Dialysate, Lactate, Low dialysate flow rate, Potassium

## Abstract

**Background:**

Observational studies of hemodialysis patients treated thrice weekly have shown that serum and dialysate potassium and bicarbonate concentrations are associated with patient outcomes. The effect of more frequent hemodialysis on serum potassium and bicarbonate concentrations has rarely been studied, especially for treatments at low dialysate flow rate.

**Methods:**

These post-hoc analyses evaluated data from patients who transferred from in-center hemodialysis (HD) to daily HD at low dialysate flow rates during the FREEDOM Study. The primary outcomes were the change in predialysis serum potassium and bicarbonate concentrations after transfer from in-center HD (mean during the last 3 months) to daily HD (mean during the first 3 months).

**Results:**

After transfer from in-center HD to daily HD (data from 345 patients, 51 ± 15 years of age, mean ± standard deviation), predialysis serum potassium decreased (*P* < 0.001) by approximately 0.4 mEq/L when dialysate potassium concentration during daily HD was 1 mEq/L; no change occurred when dialysate potassium concentration during daily HD was 2 mEq/L. After transfer from in-center HD to daily HD (data from 284 patients, 51 ± 15 years of age), predialysis serum bicarbonate concentration decreased (*P* = 0.0022) by 1.0 ± 3.3 mEq/L when dialysate lactate concentration was 40 mEq/L but increased (*P* < 0.001) by 2.5 ± 3.5 mEq/L when dialysate lactate concentration was 45 mEq/L. These relationships were dependent on serum potassium and bicarbonate concentrations during in-center HD.

**Conclusions:**

Control of serum potassium and bicarbonate concentrations during daily HD at low dialysate flow rates is readily achievable; the choice of dialysate potassium and lactate concentration can be informed when transfer is from in-center HD to daily HD.

**Electronic supplementary material:**

The online version of this article (10.1186/s12882-019-1450-7) contains supplementary material, which is available to authorized users.

## Background

Optimization of dialysate composition during hemodialysis (HD) has long been sought to normalize blood electrolyte and acid-base buffer concentrations and potentially improve patient outcomes. Tailoring of dialysate concentrations controls removal (or delivery) during the treatment, thereby modifying serum concentrations; however, optimal serum and dialysate concentrations of electrolytes and acid-base buffer concentrations for patients treated by HD remain incompletely defined [1–3].

Current understanding of the relationships between serum and dialysate concentrations of potassium and bicarbonate and patient outcomes derive largely from observational studies of HD patients treated thrice weekly. For example, observational studies have shown that predialysis serum potassium concentrations within a high normal range are associated with the lowest risk of all-cause mortality (study-dependent range: 4.6–5.3 mEq/L [4] or 4.0–5.5 mEq/L [5]). The importance of dialysate potassium concentration on all-cause mortality during thrice weekly HD therapy is less clear; elevated risks of all-cause mortality have been reported with high [4] or low [6, 7] dialysate potassium concentrations while others have reported no difference for dialysate potassium concentrations of 2 or 3 mEq/L after co-factor adjustment [5]. Evidence is more convincing that low dialysate potassium concentrations are associated with an elevated risk of sudden cardiac arrest [7–9], a leading cause of death in HD patients treated thrice weekly, particularly when serum potassium is low (≤5.0 mEq/L) [9]. Similarly, observational studies have shown that both high and low serum bicarbonate levels are associated with an elevated risk of mortality in HD patients treated thrice weekly [10]; however, the association of high bicarbonate levels with mortality is reduced after adjustment for differences in nutrition and inflammation [11, 12]. There are scant data regarding the effect of dialysate bicarbonate concentration on HD patient outcomes. The observational study by Tentori et al. [13] demonstrated a positive association between dialysate bicarbonate concentration and patient mortality, possibly due to exacerbation of intradialysis and postdialysis alkalosis.

There are no published data regarding comparable patient outcome relationships for patients treated using more frequent HD treatment schedules, and the optimal dialysate concentrations of electrolytes and buffers likely depend on HD treatment frequency. Recognizing their potential clinical importance, we describe in this report associations of dialysate potassium and lactate concentrations on serum levels of potassium and bicarbonate in patients who transferred from thrice-weekly, in-center HD to daily HD at low dialysate flow rates.

## Methods

Monthly biochemical laboratory data were available from 500 patients enrolled in the FREEDOM Study where a cohort of patients were treated with daily HD using the NxStage System One (NxStage Medical, Lawrence, MA) at low dialysate flow rates [14]. FREEDOM was a multicenter prospective cohort study of patients who were suitable candidates for daily HD and had Medicare as the primary payer. The primary intent-to-treat study outcome was the number of all-cause hospitalizations compared with that of a matched cohort of patients on conventional thrice weekly, in-center HD (ICHD); detailed inclusion and exclusion criteria have previously been described [14]. In the intent-to-treat patient cohort, age was 52 ± 15 years, 63% were male, 67% of the subjects were white, 30% were African-American and 97% were non-Hispanic. Clinical outcome findings from the FREEDOM Study have been previously published [15, 16].

The laboratory data available for the current study included those collected during the 3-month pre-enrollment period on ICHD and then monthly throughout the entire study; this report was restricted to only the first 3 months of data after transfer to daily HD. After study enrollment and collection of pre-enrollment data, patients were treated at home with daily HD using the NxStage System One. Patients were included in this study when the following data were available for at least one month: 1) treatment data after transfer to daily HD; 2) pre-enrollment ICHD serum potassium and bicarbonate concentrations; 3) pre-enrollment ICHD prescribed dialysate potassium and bicarbonate concentrations; 4) serum potassium or bicarbonate concentrations during daily HD; and 5) prescribed dialysate potassium and lactate concentrations during daily HD. Serum concentrations of bicarbonate were those of total carbon dioxide content; this terminology is virtually universal in the United States [17]. Patients with recorded bicarbonate concentrations < 5 mEq/L or ≥ 90 mEq/L were excluded. If more than one month of data were available during the 3 pre-enrollment months of ICHD or the first 3 months of follow-up with daily HD, the mean values of such data were used. The data reported herein were collected during years 2006–2012.

ICHD prescriptions were typical for HD patients treated in the United States. Dialysate concentrations of potassium during ICHD varied between 1 and 3 mEq/L (median of 2 mEq/L), and dialysate concentrations of bicarbonate during ICHD varied between 21 and 45 mEq/L (median of 35 mEq/L). Dialysate concentrations of potassium during daily HD were either 1 or 2 mEq/L, and dialysate concentrations of lactate during daily HD were either 40 or 45 mEq/L. If the 3-month average of the dialysate lactate concentration was ≤42.5 mEq/L, it was categorized as 40 mEq/L; otherwise, it was categorized as 45 mEq/L. Although the dialysate volume per treatment was recorded, exact session treatment times were not.

The primary outcomes were change in serum potassium and bicarbonate concentrations after transfer from ICHD to daily HD. All data were reported as mean ± standard deviation, and Pearson regression coefficients as mean ± standard error. The significance of changes in serum concentrations was assessed using a paired Student’s t-test. Relationships between changes in serum potassium and bicarbonate concentration and other variables were assessed by single and multiple variable linear regression. As previously described [14–16], written informed consent was obtained from all study participants in the FREEDOM Study, and a central or local institutional review board approved the study protocol at each investigation site.

## Results

There were 425 evaluable patients from the FREEDOM Study who transferred from ICHD to daily HD at low dialysate flow rates. Of those, 345 patients were evaluable for analyzing the change in serum potassium concentration and 284 for analyzing the change in serum bicarbonate concentration. Patient characteristics and treatment parameters during daily HD for these cohorts are summarized in Table 1. There were no substantial differences among these cohorts; further, these statistics were very similar when they were sub-categorized by dialysate potassium and bicarbonate concentrations during ICHD.

**Table 1:** Summary data for the entire cohort with evaluable data and those for analysis of changes in serum potassium and bicarbonate concentrations (Mean ± SD)

Patient characteristic or treatment parameter during daily HD	Evaluable cohort	Potassium cohort	Bicarbonate cohort
Number of Patients (N)	425	345	284
Age (years)	52 ± 15	51 ± 15	51 ± 15
Pre-Enrollment Body Weight (kg)	87.0 ± 23.8	86.5 ± 23.7	86.6 ± 23.2
Treatment Frequency per week	5.9 ± 0.2	5.9 ± 0.2	5.9 ± 0.2
Dialysate Volume per treatment (L)	22.3 ± 4.0	22.2 ± 4.0	22.1 ± 4.0
Weekly Dialysate Volume (L)	131 ± 25	131 ± 24	131 ± 24
Blood Flow Rate (mL/min)	465 ± 54	468 ± 52	469 ± 51
Dialysate Potassium Concentration (mEq/L)	1.07 ± 0.27	1.06 ± 0.24	1.07 ± 0.27
Dialysate Lactate Concentrations (mEq/L)	43.0 ± 3.1	43.0 ± 3.2	43.1 ± 2.4

### Potassium

In total, serum potassium concentrations were higher (*P* < 0.001) at 4.8 ± 0.6 mEq/L during ICHD than during daily HD at 4.4 ± 0.6 mEq/L. Table 2 shows serum potassium concentration for patients treated with various dialysate potassium concentrations during ICHD and daily HD. During ICHD, patients with higher serum potassium concentrations were generally treated with dialysate potassium concentrations of 1 mEq/L. When patients transferred from ICHD to daily HD with a dialysate potassium concentration of 1 mEq/L, serum potassium decreased by an approximately equivalent amount, independent of ICHD dialysate potassium concentration (decrease of 0.42 ± 0.78 mEq/l when ICHD dialysate potassium was 1 mEq/L, *P* < 0.001; 0.47 ± 0.61 mEq/L when ICHD dialysate potassium was 2 mEq/L, P < 0.001; 0.36 ± 0.74 mEq/l when ICHD dialysate potassium was 3 mEq/L, *P* = 0.0013). When patients transferred from ICHD to daily HD with a dialysate potassium concentration of 2 mEq/L, serum potassium did not change.

**Table 2:** Serum and dialysate potassium (K) concentrations during the FREEDOM study reported as mean ± sd

Dialysate K concentration (mEq/L)			Serum K concentration (mEq/L)	
Pre-enrollment ICHD	Daily HD	N	Pre-enrollment ICHD	Daily HD
1	1	46	5.3 ± 0.8	4.8 ± 0.7*
	2	1	5.4	5.8
2	1	229	4.8 ± 0.6	4.4 ± 0.5*
	2	11	4.4 ± 0.4	4.4 ± 0.5
3	1	49	4.4 ± 0.5	4.0 ± 0.7**
	2	9	4.2 ± 0.4	4.2 ± 0.4

Different from pre-enrollment ICHD value (*P < 0.001, **P = 0.0013)

**Fig. 1:**
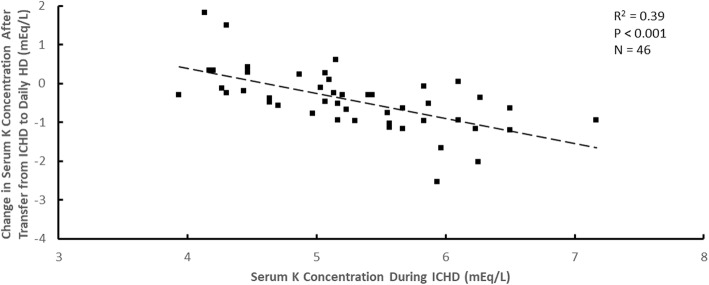
The change in serum potassium (K) concentration after transfer from ICHD to daily HD plotted versus the serum K concentration during ICHD for a dialysate K concentration during ICHD of 1 mEq/L

**Fig. 2:**
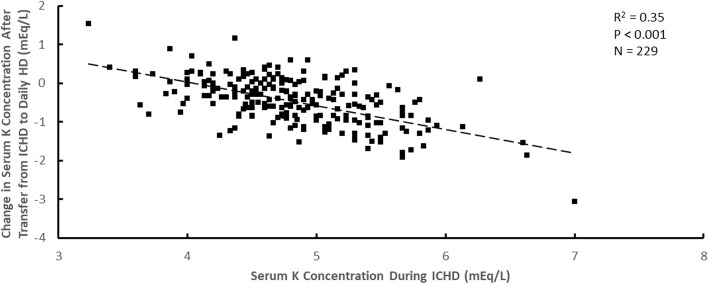
The change in serum potassium (K) concentration after transfer from ICHD to daily HD plotted versus the serum K concentration during ICHD for a dialysate K concentration during ICHD of 2 mEq/L.

**Fig. 3:**
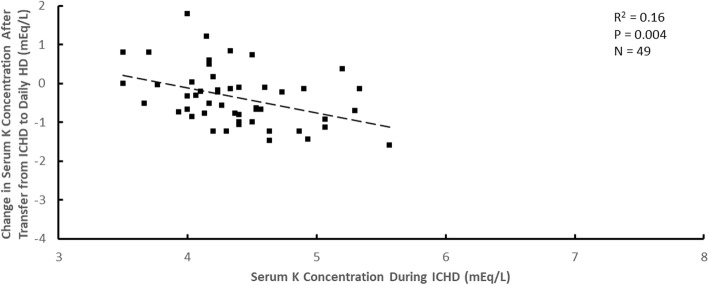
The change in serum potassium (K) concentration after transfer from ICHD to daily HD plotted versus the serum K concentration during ICHD for a dialysate K concentration during ICHD of 3 mEq/L.

Although the average decrease in serum potassium concentration after transfer to daily HD was approximately 0.4 mEq/L, the magnitude of the concentration decrease was dependent on the pre-enrollment serum potassium concentration during ICHD. The change in serum potassium concentration after transfer from ICHD to daily HD with a dialysate potassium concentration of 1 mEq/L versus the pre-enrollment serum potassium concentration during ICHD when the ICHD dialysate potassium concentration was 1 mEq/L is shown in Figs. 1 and 2 mEq/L in Figs. 2 and 3 mEq/L in Fig. 3. Although there is considerable patient-to-patient variability, the slopes of the regression lines were similar (− 0.64 ± 0.12 in Fig. 1; − 0.61 ± 0.06 in Fig. 2; − 0.65 ± 0.21 in Fig. 3). Attempts to add dialysate lactate concentration during daily HD to these relationships by multiple linear regression were not statistically significant for ICHD dialysate potassium concentrations of 1 mEq/L (*P* = 0.18), 2 mEq/L (*P* = 0.31) or 3 mEq/L (*P* = 0.44).

### Bicarbonate/lactate

In total, serum bicarbonate concentrations were 22.9 ± 3.1 mEq/L during ICHD and 24.1 ± 3.0 mEq/L during daily HD. After transfer from ICHD to daily HD, predialysis serum bicarbonate concentration decreased (*P* = 0.0022) by 1.0 ± 3.3 mEq/L when dialysate lactate concentration was 40 mEq/L but increased (*P* < 0.001) by 2.5 ± 3.5 mEq/L when dialysate lactate concentration was 45 mEq/L. These relationships were however dependent on the dialysate bicarbonate concentration during ICHD as shown in Table 3. When patients transferred from ICHD to daily HD with a dialysate lactate concentration of 40 mEq/L, serum bicarbonate decreased significantly only in patients treated during ICHD with a dialysate bicarbonate concentration of > 38 mEq/L. When patients transferred from ICHD to daily HD with a dialysate lactate concentration of 45 mEq/L, serum bicarbonate increased but not always with statistical significance. Overall, 188 (66.2%) patients achieved a serum bicarbonate concentration of ≥22 mEq/L during ICHD as recommended by KDOQI clinical practice guidelines [18]; after transfer to daily HD, the number of patients achieving or exceeding the 22 mEq/l target was higher at 224 patients (78.9%).

**Table 3:** Serum bicarbonate and dialysate bicarbonate/lactate concentrations during the FREEDOM study reported as mean ± sd

Dialysate bicarbonate/lactate concentration (mEq/L)			Serum bicarbonate concentration (mEq/L)	
Pre-enrollment ICHD (Bicarbonate)	Daily HD (Lactate)	N	Pre-enrollment ICHD	Daily HD
≤35	40	37	22.8 ± 2.4	22.5 ± 2.7
	45	83	22.1 ± 2.9	25.6 ± 3.0*
> 35 & ≤38	40	33	23.7 ± 3.1	23.2 ± 2.4
	45	41	23.8 ± 2.9	24.8 ± 2.5
> 38	40	37	23.7 ± 2.4	21.5 ± 2.5*
	45	53	22.6 ± 3.9	24.9 ± 2.7*

Different from pre-enrollment ICHD value (*P < 0.001)

**Fig. 4:**
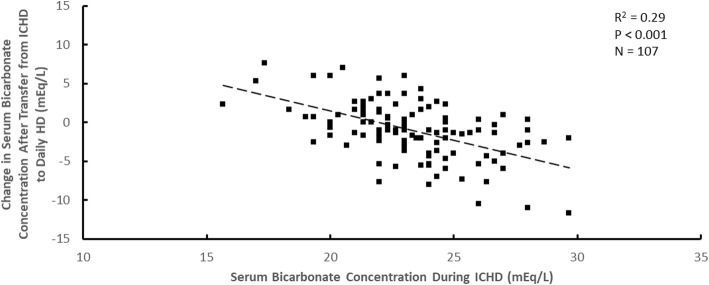
The change in serum bicarbonate concentration after transfer from ICHD to daily HD plotted versus the serum bicarbonate concentration during ICHD for a dialysate lactate concentration during daily HD of 40 mEq/L

**Fig. 5:**
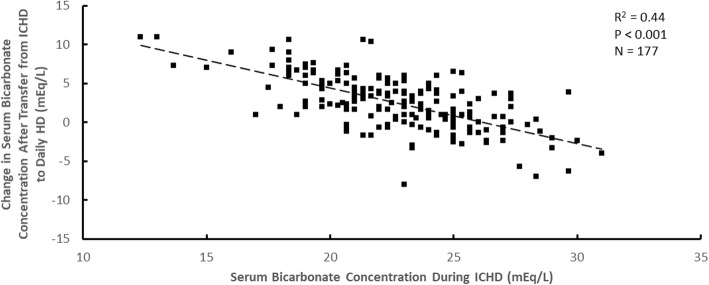
The change in serum bicarbonate concentration after transfer from ICHD to daily HD plotted versus the serum bicarbonate concentration during ICHD for a dialysate lactate concentration during daily HD of 45 mEq/L

The change in the serum bicarbonate concentration after transfer from ICHD to daily HD was also dependent on the pre-enrollment serum bicarbonate concentration during ICHD. This dependence is shown for dialysate lactate concentrations during daily HD of 40 mEq/L in Fig. 4 and 45 mEq/L in Fig. 5. The slopes of these two graphs are similar, the lines being displaced vertically. For all data together, the best fit regression equation for the change in serum bicarbonate after transfer (Δ[serum bicarbonate]) was

$$ \Delta \left[\mathrm{serum}\ \mathrm{bicarbonate}\right]=-7.135+0.583\times \left[\mathrm{dialysate}\ \mathrm{lactate}\ \mathrm{during}\ \mathrm{daily}\ \mathrm{HD}\right]-0.734\ \left[\mathrm{serum}\ \mathrm{bicarbonate}\ \mathrm{during}\ \mathrm{ICHD}\right] $$

All concentrations in the above equation are in mEq/L. This multivariate regression equation is of high statistical significance (*P* < 0.001), yet its predictability is relatively low for individual patients (R^2^ = 0.53).

## Discussion

The current report describes the effect of dialysate potassium and lactate concentrations on serum concentrations of potassium and bicarbonate after transfer from ICHD to daily HD at low dialysate flow rates. With respect to potassium, prior clinical experience [19, 20] and theoretical models [21] have reported minimal changes in serum potassium concentration after transfer from ICHD to daily HD; however, none of the those reports were for patients treated with daily HD at low dialysate flow rates. Clinical experience reporting serum potassium concentrations in patients treated by daily HD at low dialysate flow rates have been previously reported in two studies. King and Glickman reported anecdotally that such patients treated with a dialysate potassium concentration of 1 mEq/L achieved a serum potassium concentration of 4.5 ± 0.5 mEq/L, with 83% of measured values between 3.5 and 5.2 mEq/L [22]. Recently, Cherukuri et al. reported that serum potassium decreased significantly from 4.80 ± 0.63 mEq/l to 4.59 ± 0.78 mEq/L after 6 months of daily HD in a cohort of 104 patients treated with similar prescriptions to those in the FREEDOM Study (although the dialysate potassium concentration prescribed was not reported) [23]. The current observations are consistent with those observed previously.

It has been previously reported [20], and confirmed by theoretical models [21], that intradialytic reductions in serum potassium concentration are smaller during daily HD than thrice-weekly ICHD; however, none of those reports considered daily HD prescriptions at low dialysate flow rates. Data collected during the FREEDOM Study did not include measurements of postdialysis potassium concentrations; thus, the current study cannot empirically confirm that similar results will pertain to daily HD at low dialysate flow rates. As outlined in the (Additional file 1: Potassium Modeling), however, we have modified a mathematical model of potassium kinetics during HD [24] to include colonic clearance of potassium and have used that model to predict the effect of dialysate potassium concentration on predialysis serum potassium concentration, postdialysis serum potassium concentration and the intradialytic reduction in serum potassium concentration. In attempting to generalize the reported results from the FREEDOM Study, we used this model to make predictions for other more frequent HD prescriptions with treatment frequencies between 3.5 and 6 times per week with dialysate volumes of 20–60 L per treatment session after transfer from ICHD with a dialysate potassium concentration of 2 mEq/L. Theoretical model simulations indicated that changes in predialysis, postdialysis and intradialytic reduction in serum potassium concentration were primarily a function of total dialysate volume per week under such conditions (see Table 4). Regarding decreases in predialysis serum potassium concentration, the theoretical model predicts that total weekly dialysate volumes of approximately 120 L per week will decrease predialysis serum potassium concentrations when using a dialysate potassium concentration of 1 mEq/L during daily HD but not when using one of 2 mEq/L, predictions that are consonant with empirical findings from the FREEDOM Study reported here. These model predictions also suggest that approximately 120 L of total weekly dialysate volume during daily HD will not result in substantial reductions in postdialysis serum potassium concentration nor increases in the intradialytic reduction in serum potassium concentration. It should be emphasized that these model predictions have not been validated using clinical data and should only be considered hypothesis generating.

**Table 4:** Range of total dialysate volume per week during more frequent HD (MFHD) using a dialysate potassium concentration of 1 or 2 mEq/L require to decrease the predialysis and postdialysis potassium concentration and increase intradialytic reduction in potassium concentration after transfer from ICHD using a dialysate potassium concentration of 2 mEq/L

	Total dialysate volume per week to change potassium concentration		
MFHD dialysate potassium concentration	Decrease predialysis	Decrease postdialysis	Increase intradialytic reduction
1 mEq/L	80-120 L	120–200 L	> 360 L
2 mEq/L	120–175 L	300 L	> 360 L

With respect to bicarbonate, daily HD at conventional dialysate flow rates was shown to not alter predialysis nor postdialysis serum bicarbonate concentrations (using dialysis solutions containing bicarbonate as buffer) [19]. King and Glickman reported that the serum bicarbonate concentration of patients treated by daily HD at low dialysate flow rates using the NxStage System One was 23.9 ± 1.6 mEq/L [22]. Seventeen of those patients were prescribed a dialysate lactate concentration of 45 mEq/L; only one was prescribed a dialysate lactate concentration of 40 mEq/L. Cherukuri et al., in the same study noted above [23], showed that serum bicarbonate concentrations increased significantly from 23.1 ± 3.5 mEq/L to 24.1 ± 2.9 mEq/L after 6 months of daily HD at low dialysate flow rates (the dialysate lactate concentrations prescribed were not however reported). The current observations are comparable to those limited data. Although it is expected that predialysis serum bicarbonate concentrations will be higher during daily HD than ICHD because of the shorter interdialytic interval (assuming constant daily intake of protein and buffer base transferred to the patient) [25], a larger number of patients treated with daily HD in the current study achieved the KDOQI-recommended predialysis serum bicarbonate concentration of ≥22 mEq/L than when these same patients were treated with ICHD. Although this observation appears to be an advantage of daily HD at low dialysate flow rates, additional studies related to patient outcomes are needed to define optimal predialysis serum bicarbonate concentrations.

Although the current study reports novel data on patients treated by daily HD at low dialysate flow rates, there are several limitations to its interpretation and generalizability. First, the data collected were limited. As mentioned above, postdialysis serum concentrations were not measured; therefore, the effect of transfer from ICHD to daily HD on postdialysis and intradialytic changes in serum potassium concentrations could only be predicted using a mathematical model. The importance of low postdialysis serum potassium concentrations on patient mortality remains unclear. A very recent observational study demonstrated an association between low postdialysis serum potassium concentration and mortality only without adjusting for predialysis serum potassium concentration; however, the combination of both low predialysis and postdialysis serum potassium concentrations was associated with a high mortality risk [26]. Also, treatment times during daily HD were not recorded; however, high blood flow rates and dialysate volumes per session between 20 and 25 L were almost universally prescribed, indicating that the vast majority of treatments times was likely between 120 and 210 min. Second, there were limited data available for patients who were treated by daily HD using a dialysate potassium concentration of 2 mEq/L. This was because such dialysis solutions were only commercially available during the latter part of the FREEDOM Study. Finally, the effect of differences in serum potassium and bicarbonate concentrations and dialysate potassium and lactate concentrations during daily HD at low dialysate flow rates on patient outcomes was not possible using the available data; however, the design of future definitive studies on patient outcomes might be made possible based on the results of the current study.

## Conclusions

Control of serum potassium and bicarbonate concentrations during daily HD at low dialysate flow rates is readily achievable; the choice of the dialysate potassium and lactate concentration can be informed when transfer is from ICHD to daily HD.

## Additional files

Additional file 1: Potassium Modeling During MFHD. This additional file describes and validates a new mathematical model of potassium kinetics during more frequent hemodialysis. (DOCX 108 kb)

Additional file 2: Full List of Investigation Sites. This additional file provides a full list of the investigation sites who participated in the FREEDOM Study. (DOCX 17 kb)

## Data Availability

The datasets used and/or analyzed during the current study are available from the corresponding author on reasonable request.

## Abbreviations

FREEDOM: Following Rehabilitation Economics and Every-Day Dialysis Outcome Measurements
HD: Hemodialysis
ICHD: In-center hemodialysis
K: Potassium
KDOQI: Kidney Disease Outcomes Quality Initiative (National Kidney Foundation)
MFHD: More frequent hemodialysis
SD: Standard deviation
