# Supplementary material for: Effect of dialysate potassium and lactate on serum potassium and bicarbonate concentrations during daily hemodialysis at low dialysate flow rates
Source: BMC Nephrol. 2019 Jul 9;20:252. doi: 10.1186/s12882-019-1450-7 (PMC6617706; doi:10.1186/s12882-019-1450-7)
Supplement: Supplementary file 1 — Potassium Modeling During MFHD. This additional file describes and validates a new mathematical model of potassium kinetics during more frequent hemodialysis. (DOCX 108 kb) [file 12882_2019_1450_MOESM1_ESM.docx]

**Potassium Modeling During MFHD**

**Supplemental Material For:**

**Manuscript Title: Effect of Dialysate Potassium and Lactate on Serum Potassium and Bicarbonate Concentrations During Daily Hemodialysis at Low Dialysate Flow Rates**

Authors: Leypoldt JK, Kraus MA, Jaber BL, Weinhandl ED, Collins AJ

**Model Development/Validation**

A pseudo one-compartment model described by Agar et al^1^ was modified to include potassium colonic clearance^2-4^ to better describe the kinetics of potassium during hemodialysis (HD); this modification required the addition of a non-kidney clearance pathway for removal of potassium from the central compartment of the model (K_NK_) as illustrated in Figure S1. For this modified model, the dependence of serum potassium concentration in the central compartment (C) as a function of time during a given treatment is described by the following equation

| $\frac{C\left( t \right)}{C_{\mathrm{pre}}}=\frac{K_{M}+\frac{DC_{d}}{C_{\mathrm{pre}}}}{K_{M}+K_{K}+K_{\mathrm{NK}}+D-Q_{f}}+\left[ 1-\frac{K_{M}+\frac{DC_{d}}{C_{\mathrm{pre}}}}{K_{M}+K_{K}+K_{\mathrm{NK}}+D-Q_{f}} \right]\times\left[ \frac{V(t)}{V_{\mathrm{pre}}} \right]^{\frac{K_{M}+K_{K}+K_{\mathrm{NK}}+D-Q_{f}}{Q_{f}}}$ | (S1) |
| --- | --- |

where C_pre_ denotes predialysis serum potassium concentration, C_d_ denotes dialysate concentration of potassium, K_M_ denotes potassium mobilization clearance, K_K_ denotes residual kidney clearance of potassium, Q_f_ denotes the ultrafiltration rate during the treatment (assumed constant), D denotes dialysance of potassium for the dialyzer, V_pre_ denotes volume of the central compartment at the beginning of the treatment, and V(t) denotes linear reduction of the central compartment volume with time during the treatment. This model was first validated by comparing average kinetic modeling data collected during the HEMO Study^1^ with model predictions assuming a postdialysis central compartment volume of 20 L and a treatment time of 220 min. The values of potassium mobilization clearance and dialysance of potassium were assumed equal to average values estimated previously that were dependent on the prescribed dialysate potassium concentration.^1^

Figure S2 shows a comparison of postdialysis potassium concentrations measured during the HEMO Study and those predicted by the model (note that the measured and model predialysis potassium concentrations were assumed equal) ; graphs are shown independently for dialysate potassium concentrations of 1 mEq/L (A), 2 mEq/L (B) and 3 mEq/L (C). Here, kidney clearance of potassium was assumed to be negligibly small, and non-kidney clearance of potassium was assumed to be 20 mL/min. There is excellent agreement between the measured postdialysis potassium concentrations and those predicted by the modified pseudo one-compartment model.

To examine whether a modified pseudo one-compartment model is also consistent with average data from the FREEDOM Study (see main text), we next compared predialysis potassium concentrations during thrice weekly, in-center HD (ICHD) and during daily HD after transfer from ICHD (note that the measured and model predialysis potassium concentrations during ICHD were assumed equal). Here, a steady state mass balance model was assumed to be valid where the dietary intake (generation rate or G) of potassium remained the same during both ICHD and daily HD and the hemodialysis treatments were assumed to be applied symmetrically during the week.^5,6^ Under these conditions, the potassium generation rate can first be calculated by the following equation

| $G=C_{\mathrm{pre}}\frac{\left( D+K_{K}+K_{\mathrm{NK}} \right)T\bar{C}_{T}+(K_{K}+K_{\mathrm{NK}})\theta\bar{C}_{\theta}+\frac{DC_{d}T}{C_{\mathrm{pre}}}}{T+\theta}$ | (S2) |
| --- | --- |

where

| $\bar{C}_{T}=\frac{K_{M}}{K_{M}+K_{K}+K_{\mathrm{NK}}+D-Q_{f}}+\left[ 1-\frac{K_{M}}{K_{M}+K_{K}+K_{\mathrm{NK}}+D-Q_{f}} \right]\times\left[ \frac{V_{pre/T}}{K_{M}+K_{K}+K_{\mathrm{NK}}+D} \right]\times\left( 1-\left[ \frac{V_{\mathrm{post}}}{V_{\mathrm{pre}}} \right]^{\frac{K_{M}+K_{K}+K_{\mathrm{NK}}+D}{Q_{f}}} \right)$ | (S3) |
| --- | --- |

and

| $\bar{C}_{\theta}=\frac{K_{M}}{K_{M}+K_{K}+K_{\mathrm{NK}}+W_{g}}+\left[ \frac{C_{\mathrm{post}}}{C_{\mathrm{pre}}}-\frac{K_{M}}{K_{M}+K_{K}+K_{\mathrm{NK}}+W_{g}} \right]\times\left[ \frac{V_{\mathrm{post}}/\theta}{K_{M}+K_{K}+K_{\mathrm{NK}}} \right]\times\left( 1-\left[ \frac{V_{\mathrm{pre}}}{V_{\mathrm{post}}} \right]^{-\frac{K_{M}+K_{K}+K_{\mathrm{NK}}}{W_{g}}} \right)$ | (S4) |
| --- | --- |

where W_g_ denotes the rate of weight gain during the interdialytic interval, θ denotes the interdialytic interval, and the subscripts _pre_ and _post_ denote predialysis and postdialysis values. All parameters in equations (S2)-(S4) are those during ICHD. Then, equation (S2) can be rearranged to yield the following equation for calculating the new predialysis serum potassium concentration during daily HD (C_pre_^Daily HD^) after transfer from ICHD as

| $C_{\mathrm{pre}}^{Daily HD}=\frac{G\times(T+\theta)}{\left( D+K_{K}+K_{\mathrm{NK}} \right)T\bar{C}_{T}+(K_{K}+K_{\mathrm{NK}})\theta\bar{C}_{\theta}+\frac{DC_{d}T}{C_{\mathrm{pre}}^{Daily HD}}}$ | (S5) |
| --- | --- |

Here, equations (S3)-(S5) are now calculated using only the treatment parameters during daily HD but with G determined as described above in equation (S2); note that equation (S5) must be solved implicitly for C_pre_^Daily HD^.

Patient and prescription parameters assumed in all subsequent predictions or simulations are shown in Table S1; these parameters were chosen based on the previous publication by Agar et al^1^ or mean values obtained during the FREEDOM Study as described in the main text. The potassium colonic clearance (K_NK_) was set equal to 20 mL/min and the potassium mobilization clearance was assumed dependent on predialysis potassium concentration (C_pre_) as given by (K_M_ = 200 mL/min during ICHD and K_M_ = 300 mL/min during daily HD). Larger values of K_M_ were assumed during daily HD because K_M_ was previously shown to be larger for lower predialysis serum potassium concentrations.^1^ Residual kidney clearance of potassium was assumed to be negligibly small. The model was also used to predict postdialysis serum potassium concentrations during the FREEDOM Study.

Figure S3 compares predialysis serum potassium concentrations measured during the FREEDOM Study and those predicted by the model for patients prescribed a dialysate potassium concentration of 1 mEq/L during ICHD and either 1 or 2 mEq/L during daily HD. Also shown are postdialysis potassium concentrations predicted by the model. Figures S4 and S5 show similar predictions for patients prescribed a dialysate potassium concentration of 2 mEq/L and 3 mEq/L during ICHD, respectively. There is reasonable agreement between predialysis serum potassium concentrations and those predicted by the model; the best agreement is among data where the dialysate potassium concentration during ICHD was 2 mEq/L, where the sample size was largest. This agreement suggests that the postdialysis potassium concentrations can be used to estimate intradialytic reductions in serum potassium concentrations under these conditions; those values are tabulated in Table S2. It can be readily observed that intradialytic reductions in serum potassium concentrations are substantially less during daily HD than during ICHD.

**Further Model Predictions**

The modified pseudo one-compartment model can then be generalized to predict postdialysis potassium concentrations for arbitrary more frequent HD prescriptions. After examining a variety of predictions, it was evident that the change in predialysis and postdialysis serum concentrations of potassium after transfer from ICHD to more frequent HD were largely determined by the total dialysate volume per week, not on the precise hemodialysis prescription. Those predictions are summarized in Table 4 of the main text.

**Table S1**

Patient and HD Prescription Parameters Assumed in All Simulations (ICHD denotes thrice-weekly, in-center HD)

| Parameter | Value |
| --- | --- |
| Potassium central distribution volume | 20 L |
| Weekly fluid removal | 12 L |
| *During ICHD* |  |
| Blood flow rate | 400 mL/min |
| Dialysate flow rate | 500 mL/min |
| Treatment time | 220 min |
| Dialyzer K_o_A for potassium | 383 mL/min |
| *During Daily HD* |  |
| Blood flow rate | 450 mL/min |
| Dialysate volume per treatment | 22 L |
| Treatment time | 180 min |
| Dialyzer K_o_A for potassium | 213 mL/min |

**Table S2**

Intradialytic Reduction in Serum Potassium Concentration Predicted by a Modified Pseudo One-Compartment Model

| Dialysate Potassium Concentration (mEq/) | | Intradialytic Reduction in Serum Potassium Concentration (mEq/L) | |
| --- | --- | --- | --- |
| During ICHD | During Daily HD | During ICHD | During Daily HD |
| 1 | 1 | 2.00 | 1.03 |
|  | 2 | 2.05 | 0.89 |
| 2 | 1 | 1.31 | 0.86 |
|  | 2 | 1.13 | 0.61 |
| 3 | 1 | 0.67 | 0.72 |
|  | 2 | 0.57 | 0.50 |

Figure S1 - Diagrammatic representation of the modified pseudo one-compartment model that describes changes in potassium concentration (C) within the central compartment volume (V). The pathways of potassium transport include mobilization from the peripheral compartment or K_M_ × (C_pre_ – C) where C_pre_ denotes the predialysis concentration of potassium, removal by the dialyzer or D × (C – C_d_) where C_d_ denotes dialysate concentration of potassium, removal by residual kidney function or K_K_ × C and removal by non-kidney mechanisms or K_NK_ × C.


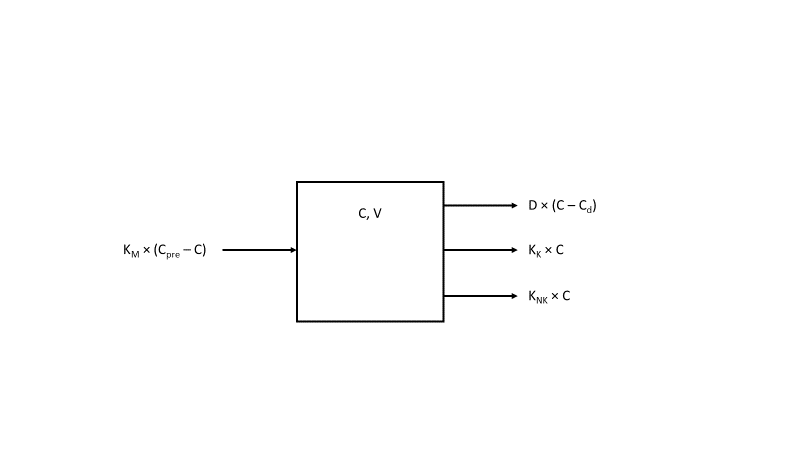


Figure S2A – Predialysis and postdialysis potassium concentrations measured during the HEMO Study and predictions from a modified pseudo one-compartment model of potassium kinetics. The prescribed dialysate potassium concentration was 1 mEq/L during the treatments, and the potassium mobilization clearance was assumed as 177 mL/min.^1^


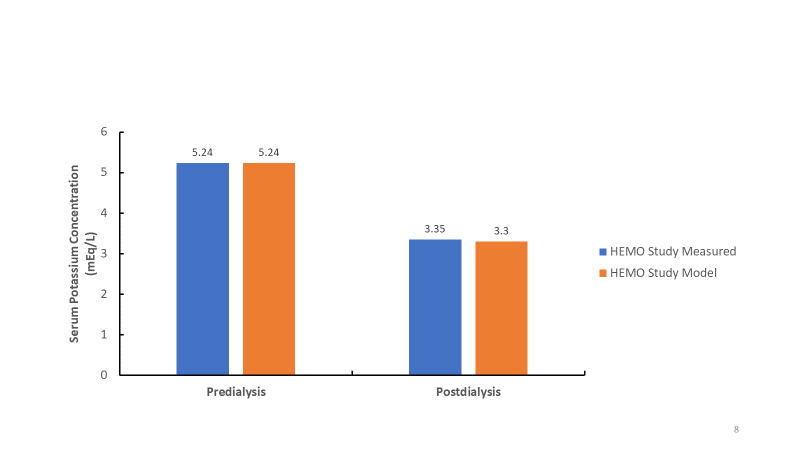


Figure S2B – Predialysis and postdialysis potassium concentrations measured during the HEMO Study and predictions from a modified pseudo-one compartment model of potassium kinetics. The prescribed dialysate potassium concentration was 2 mEq/L during the treatments, and the potassium mobilization clearance was assumed as 152 mL/min.^1^


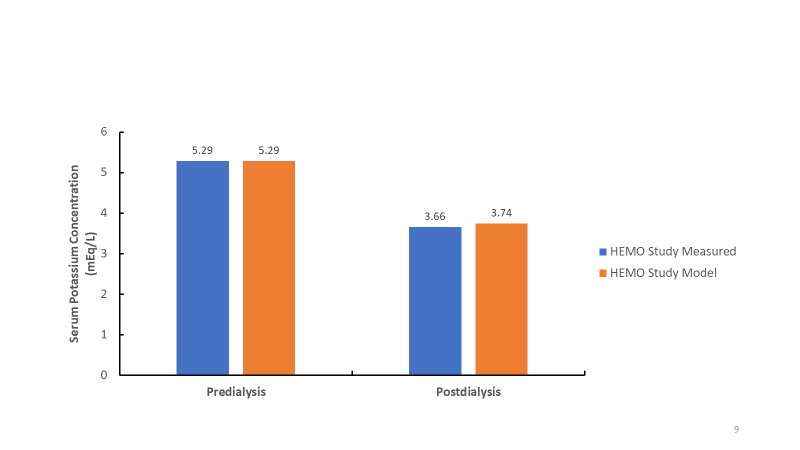


Figure S2C – Predialysis and postdialysis potassium concentrations measured during the HEMO Study and predictions from a modified pseudo one-compartment model of potassium kinetics. The prescribed dialysate potassium concentration was 3 mEq/L during the treatments, and the potassium mobilization clearance was assumed as 161 mL/min.^1^


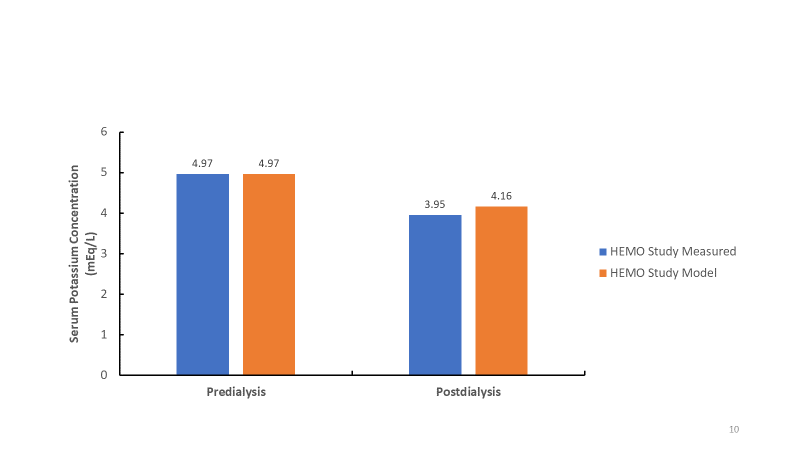


Figure S3 – Predialysis potassium concentrations during ICHD and daily HD after transfer from ICHD that were measured during the FREEDOM Study and predictions from a modified pseudo one-compartment model of potassium kinetics. Also shown are the postdialysis potassium concentrations predicted by the model. The prescribed dialysate potassium concentration was 1 mEq/L during ICHD, and either 1 mEq/L or 2 mEq/L during daily HD.


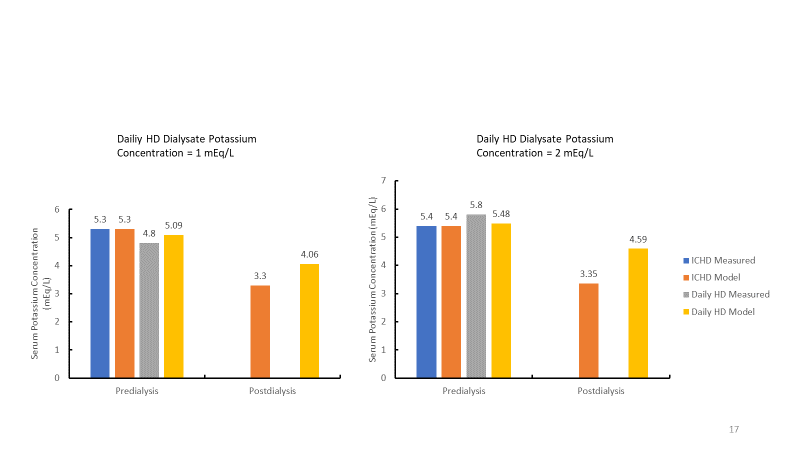


Figure S4 – Predialysis potassium concentrations during ICHD and daily HD after transfer from ICHD that were measured during the FREEDOM Study and predictions from a modified pseudo one-compartment model of potassium kinetics. Also shown are the postdialysis potassium concentrations predicted by the model. The prescribed dialysate potassium concentration was 2 mEq/L during ICHD, and either 1 mEq/L or 2 mEq/L during daily HD.


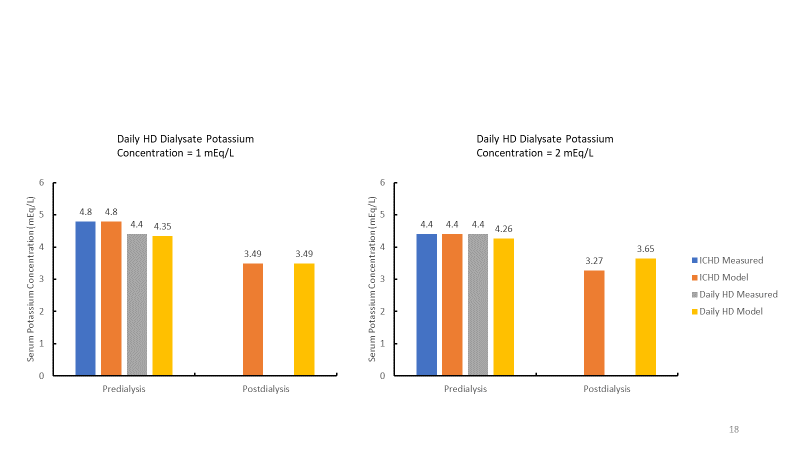


Figure S5 – Predialysis potassium concentrations during ICHD and daily HD after transfer from ICHD that were measured during the FREEDOM Study and predictions from a modified pseudo one-compartment model of potassium kinetics. Also shown are the postdialysis potassium concentrations predicted by the model. The prescribed dialysate potassium concentration was 3 mEq/L during ICHD, and either 1 mEq/L or 2 mEq/L during daily HD.


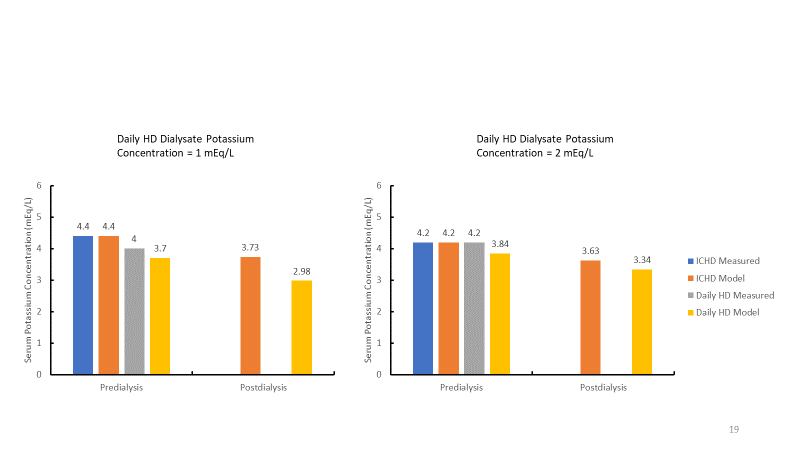


**REFERENCES**

1. Agar BU, Culleton BF, Fluck R, Leypoldt JK. Potassium kinetics during hemodialysis. *Hemodial Int* 2015; **19**(1): 23-32.

2. Hayes CP, Jr., McLeod ME, Robinson RR. An extravenal mechanism for the maintenance of potassium balance in severe chronic renal failure. *Trans Assoc Am Physicians* 1967; **80**: 207-16.

3. Hayes CP, Jr., Robinson RR. Fecal Potassium Excretion in Patients on Chronic Intermittent Hemodialysis. *Trans Am Soc Artif Intern Organs* 1965; **11**: 242-6.

4. Rachoin JS, Weisberg LS. How should dialysis fluid be individualized for the chronic hemodialysis patient? Potassium. *Semin Dial* 2008; **21**(3): 223-5.

5. Leypoldt JK, Agar BU, Akonur A, Gellens ME, Culleton BF. Steady state phosphorus mass balance model during hemodialysis based on a pseudo one-compartment kinetic model. *Int J Artif Organs* 2012; **35**(11): 969-80.

6. Leypoldt JK, Agar BU, Bernardo AA, Culleton BF. Prescriptions of dialysate potassium concentration during short daily or long nocturnal (high dose) hemodialysis. *Hemodial Int* 2016; **20**(2): 218-25.
