# Supplementary material for: Effect of dialysate potassium and lactate on serum potassium and bicarbonate concentrations during daily hemodialysis at low dialysate flow rates
Source: BMC Nephrol. 2019 Jul 9;20:252. doi: 10.1186/s12882-019-1450-7 (PMC6617706; doi:10.1186/s12882-019-1450-7)
Supplement: Supplementary file 2 — Full List of Investigation Sites. This additional file provides a full list of the investigation sites who participated in the FREEDOM Study. (DOCX 17 kb) [file 12882_2019_1450_MOESM2_ESM.docx]

**Full List of Investigation Sites**

**Supplementary Material For:**

**Manuscript Title: Effect of Dialysate Potassium and Lactate on Serum Potassium and Bicarbonate Concentrations During Daily Hemodialysis at Low Dialysate Flow Rates**

Authors: Leypoldt JK, Kraus MA, Jaber BL, Weinhandl ED, Collins AJ

The following is a full list of the 41 investigation sites who participated in the FREEDOM Study.

| **United States, Arkansas** | |
| --- | --- |
| Fort Smith Regional Dialysis Center |  |
| Fort Smith, Arkansas, United States, 72901 | |
| Arkansas Nephrology Research Associates |  |
| Hot Springs, Arkansas, United States, 71901 | |
| **United States, California** | |
| Renal Advantage (RAI) |  |
| Garden Grove, California, United States, 92843 | |
| Satellite Healthcare/Wellbound |  |
| Mountain View, California, United States, 94041 | |
| Renal Advantage (RAI) |  |
| Oakland, California, United States, 94612 | |
| Kidney Center, Inc. |  |
| Simi Valley, California, United States, 93065 | |
| **United States, Colorado** | |
| University of Colorado Hospital |  |
| Aurora, Colorado, United States, 80045 | |
| **United States, Connecticut** | |
| Metabolism Associates - New Haven CAPD |  |
| New Haven, Connecticut, United States, 06511 | |
| **United States, Florida** | |
| RAI Palm Harbor, Outcomes Research Int'l |  |
| Hudson, Florida, United States, 34667 | |
| **United States, Georgia** | |
| Emory, Renal Care Partners of Dunwoody |  |
| Sandy Springs, Georgia, United States, 30350 | |
| **United States, Illinois** | |
| Circle Medical Management |  |
| Chicago, Illinois, United States, 60607 | |
| Affiliated Home Dialysis |  |
| Glen Ellyn, Illinois, United States, 60137 | |
| **United States, Indiana** | |
| Indiana University Medical Center |  |
| Indianapolis, Indiana, United States, 46202 | |
| Nephrology, Inc. |  |
| Mishawaka, Indiana, United States, 46545 | |
| **United States, Kansas** | |
| Kansas Dialysis Services |  |
| Topeka, Kansas, United States, 66606 | |
| **United States, Kentucky** | |
| University of Louisvile KDP |  |
| Louisville, Kentucky, United States, 40202 | |
| **United States, Massachusetts** | |
| Dialysis at Home, Inc |  |
| Brighton, Massachusetts, United States, 02135 | |
| **United States, Michigan** | |
| Great Lakes Renal Network |  |
| Alma, Michigan, United States, 48801 | |
| Henry Ford Health System |  |
| Detroit, Michigan, United States, 48202 | |
| Munson Dialysis Center |  |
| Traverse City, Michigan, United States, 49684 | |
| Renal Advantage (RAI) |  |
| Wyoming, Michigan, United States, 49509 | |
| **United States, Minnesota** | |
| Mayo Clinic |  |
| Rochester, Minnesota, United States, 55905 | |
| **United States, Missouri** | |
| NRA Home |  |
| Frontenac, Missouri, United States, 63131 | |
| Washington University/Barnes Jewish Dialysis Center |  |
| St. Louis, Missouri, United States, 63108 | |
| **United States, Nebraska** | |
| Dialysis Center of Lincoln |  |
| Lincoln, Nebraska, United States, 66510 | |
| Renal Advantage |  |
| Omaha, Nebraska, United States, 68198 | |
| **United States, New Jersey** | |
| Silver Care Center |  |
| Cherry Hill, New Jersey, United States, 07675 | |
| Lillian Booth Dialysis Center |  |
| Westwood, New Jersey, United States, 07675 | |
| **United States, New York** | |
| Apollo Healthcare - Niagara Renal Center |  |
| Niagara Falls, New York, United States, 14304 | |
| Hortense and Louis Rubin Dialysis Center |  |
| Saratoga Springs, New York, United States, 12866 | |
| **United States, North Carolina** | |
| Wake Forest University/Piedmont Dialysis |  |
| Winston-Salem, North Carolina, United States, 27157 | |
| **United States, Ohio** | |
| Community Physicians Dialysis Centers |  |
| Springfield, Ohio, United States, 45505 | |
| **United States, Pennsylvania** | |
| Wellspan Dialysis |  |
| York, Pennsylvania, United States, 15198 | |
| **United States, South Carolina** | |
| Renal Advantage (RAI) |  |
| Charleston, South Carolina, United States, 29414 | |
| **United States, Tennessee** | |
| Chattanooga Kidney Center |  |
| Chattanooga, Tennessee, United States, 37421 | |
| **United States, Texas** | |
| University of Texas Medical Branch |  |
| Galveston, Texas, United States, 77555 | |
| Barlite SW Kidney Center |  |
| San Antonio, Texas, United States, 78224 | |
| **United States, Virginia** | |
| Renal Advantage (RAI) |  |
| Hampton, Virginia, United States, 23666 | |
| Virginia Commonwealth University/Medical College of Virginia |  |
| Richmond, Virginia, United States, 23298 | |
| **United States, Washington** | |
| Northwest Kidney Centers |  |
| Seattle, Washington, United States, 98122 | |
| **United States, Wisconsin** | |
| Commonwealth Dialysis |  |
| Greenfield, Wisconsin, United States, 53220 | |
